# Nerve growth factor promotes differentiation and protects the oligodendrocyte precursor cells from *in vitro* hypoxia/ischemia

**DOI:** 10.3389/fnins.2023.1111170

**Published:** 2023-02-16

**Authors:** Vito Antonio Baldassarro, Maura Cescatti, Maria Luisa Rocco, Luigi Aloe, Luca Lorenzini, Luciana Giardino, Laura Calzà

**Affiliations:** ^1^Department of Veterinary Medical Science, University of Bologna, Bologna, Italy; ^2^IRET Foundation, Bologna, Italy; ^3^Health Science and Technologies - Interdepartmental Center for Industrial Research (HST-ICIR), University of Bologna, Bologna, Italy; ^4^Department of Pharmacy and Biotechnology, University of Bologna, Bologna, Italy; ^5^Montecatone Rehabilitation Institute, Bologna, Italy

**Keywords:** nerve growth factor, oligodendrocyte precursor cells, hypoxia/ischemia, oxygen and glucose deprivation (OGD), developmental myelination, remyelination

## Abstract

**Introduction:**

Nerve growth factor (NGF) is a pleiotropic molecule acting on different cell types in physiological and pathological conditions. However, the effect of NGF on the survival, differentiation and maturation of oligodendrocyte precursor cells (OPCs) and oligodendrocytes (OLs), the cells responsible for myelin formation, turnover, and repair in the central nervous system (CNS), is still poorly understood and heavily debated.

**Methods:**

Here we used mixed neural stem cell (NSC)-derived OPC/astrocyte cultures to clarify the role of NGF throughout the entire process of OL differentiation and investigate its putative role in OPC protection under pathological conditions.

**Results:**

We first showed that the gene expression of all the neurotrophin receptors (*TrkA*, *TrkB*, *TrkC*, and *p75^NTR^*) dynamically changes during the differentiation. However, only *TrkA* and *p75^NTR^* expression depends on T3-differentiation induction, as *Ngf* gene expression induction and protein secretion in the culture medium. Moreover, in the mixed culture, astrocytes are the main producer of NGF protein, and OPCs express both *TrkA* and *p75^NTR^*. NGF treatment increases the percentage of mature OLs, while NGF blocking by neutralizing antibody and TRKA antagonist impairs OPC differentiation. Moreover, both NGF exposure and astrocyte-conditioned medium protect OPCs exposed to oxygenglucose deprivation (OGD) from cell death and NGF induces an increase of AKT/pAKT levels in OPCs nuclei by TRKA activation.

**Discussion:**

This study demonstrated that NGF is implicated in OPC differentiation, maturation, and protection in the presence of metabolic challenges, also suggesting implications for the treatment of demyelinating lesions and diseases.

## 1. Introduction

Nerve growth factor (NGF) is a neurotrophic factor discovered in 1950 for its properties of promoting the growth and survival of peripheral sensory and sympathetic nerve cells (Levi-Montalcini, 1987). Since its discovery, different roles of NGF in different cell types have been identified, highlighting many biological roles and potential therapeutic applications (Aloe et al., 2015), ultimately leading to the definition of NGF as a “pleiotropic molecule” (Ransohoff and Trebst, 2000). A possible role of NGF in developmental myelination and myelin repair in adulthood has been hypothesized for both the central and the peripheral nervous system, considering axons and myelinating cells, i.e., Schwann cells in the peripheral nervous system and oligodendrocyte precursor cells (OPCs) in the central nervous system (CNS) as NGF targets (Althaus et al., 2008; Webber and Zochodne, 2010), but results are contrasting and heavily debated.

Oligodendrocyte precursor cells (OPCs), as recognized by the heterogeneous neural/glial antigen 2 or NG2 chondroitin sulfate proteoglycan membrane marker, or by the expression of the platelet-derived growth factor alpha receptor (PDGFαR), derive from neural stem cells (NSCs) during development. These cells migrate in three different temporal waves, being responsible for developmental myelination (Kessaris et al., 2006). In the healthy adult brain, NG2-positive OPCs represent the largest population of dividing cells (Michalski and Kothary, 2015), and are responsible for the remodeling of dynamic processes which are continuously generating new myelin (Yeung et al., 2014), and for reshaping myelin volume in an activity-dependent manner (Bengtsson et al., 2005; Schlegel et al., 2012). OPCs are also the cells responsible for the repair of damaged myelin. Indeed, this process is not finalized by pre-existing mature oligodendrocytes (OLs), but by resident precursors and OPCs newly formed by mitosis and by the asymmetric division of NSCs in the subventricular zone (SVZ). Precursors and NSCs are activated by demyelinating stimuli, then proliferating and migrating to the demyelinated axons, where they rebuild the functional myelin sheath (Fukushima et al., 2015). Indeed, remyelination is the only CNS reparative process which can lead to complete anatomical and functional regeneration (Crawford et al., 2013). Notably, this repair process in impaired in conditions characterized by intense inflammation and tissue metabolic distress, like in multiple sclerosis, brain and spinal cord traumatic lesions, myelin repair failure is considered responsible for chronic disabilities and a reliable therapeutic target (Cunniffe and Coles, 2019).

Oligodendrocyte precursor cell generation and differentiation and OL maturation during development and adulthood are finely regulated by a plethora of molecules (Bercury and Macklin, 2015). Thyroid hormones (THs), particularly the active form triiodothyronine (T3), are key factors in these regulatory processes (Billon, 2002, 2004; Calzà et al., 2018). T3 acts at genomic level, binding the thyroid hormone receptors (TRs), a class of nuclear receptors that regulate the expression of specific genes controlling the OPC cell cycle and maturation (Casaccia-Bonnefil and Liu, 2003; Baxi et al., 2014). Moreover, an intricate and still unclear network of signals involving different cell types, in particular the complex communication between axons and OPCs, is responsible for the proper functioning of this process, both during developmental myelination and myelin repair. This includes growth factors, protein kinases, and extracellular matrix molecules, which are involved in epigenetic modifications, transcriptional/translational regulation, and actin cytoskeleton arrangement (Bercury and Macklin, 2015).

Although a significant body of data suggests that neurotrophins participate in neural precursor regulation (Althaus et al., 2008), the role of NGF in OPC biology during development and in the adult brain is still widely disputed. In the present study, we investigated the role of NGF in OPC differentiation, maturation, and protection from oxygen-glucose deprivation (OGD), using OPCs derived from fetal NSCs as an *in vitro* system. This *in vitro* model replicates all stages of the oligodendrocyte differentiation process, from stem cells to mature OLs.

## 2. Materials and methods

### 2.1. Cell preparation and cultures

All protocols described herein were carried out according to the European Community Council Directives (86/609/EEC) and comply with the guidelines published in the *NIH Guide for the Care and Use of Laboratory Animals*.

**FIGURE 1:**
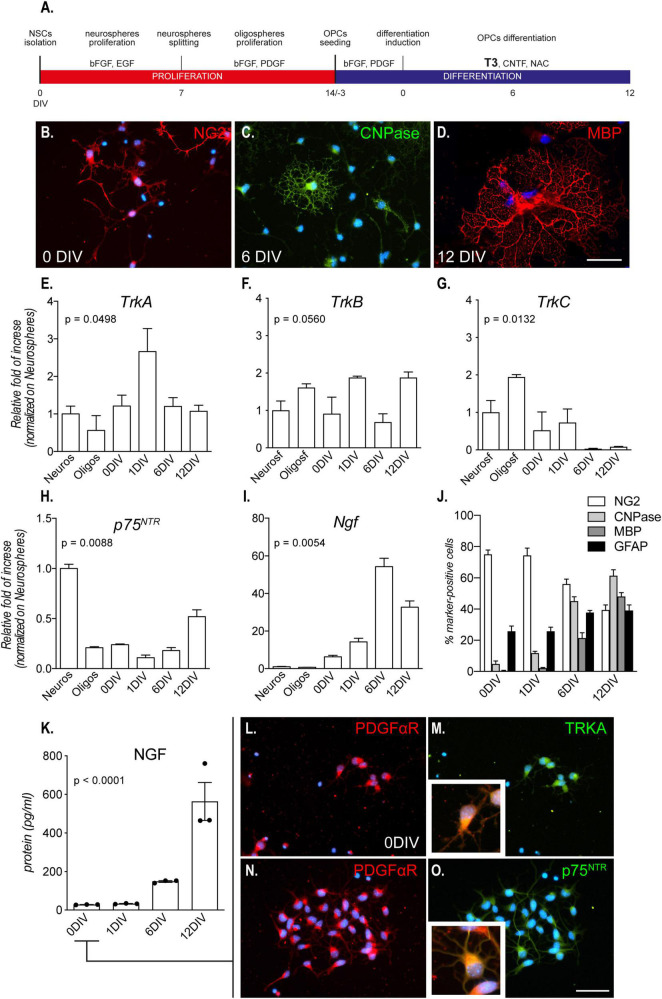
Culture characterization. **(A)** Mixed OPC/astrocyte cultures were obtained from NSC isolation and neurosphere expansion (bFGF and EGF). Following oligosphere generation by spheroid splitting and bFGF/PDGF exposure, spheres were dissociated, and single cell cultures seeded in the same medium. After 3 DIVs, the medium was replaced with differentiation medium containing T3 for 12 DIVs. **(B–D)** Representative images of culture progress from OPCs (NG2-positive cells, 0 DIV, panel **B**), through mature OLs (CNPase-positive cells, 6 DIV, C) to mature/myelinating OLs (CNPase/MBP-positive cells, 12 DIV, panel **D**). Scale bar: 20 μm. **(E–I)** Graphs show relative mRNA expression of *TrkA* **(E)**, *TrkB* **(F)**, *TrkC* **(G)**, *p75^NTR^* **(H)**, and *Ngf* **(I)** genes, throughout all culture stages and normalized on neurospheres; *n* = 3 replicates for all the presented groups. **(J)** Graph shows the culture composition along the differentiation phase (DIV 0 – 12), expressed as percentage of cells positive to NG2 (OPCs), CNPase (pre-OL), MBP (mature OLs), and GFAP (astrocytes). **(K)** Graph shows absolute NGF protein quantification (pg/ml) at all differentiation stages, from 0 DIV (OPCs/astrocytes) to 12 DIVs (mature OLs/astrocytes); *n* = 3 replicates for all the presented groups. **(L–O)** Representative images of OPCs (PDGFαR-positive cells, panel **L** and panel **N**) double positive for TRKA **(M)** and p75^NTR^ **(O)**, prior to the differentiation induction mediated by T3 (0 DIV). Scale bar: 20 μm. Bars represent mean + SEM. Statistical analysis: Kruskal-Wallis test, *p*-values are reported inside the graphs. Single dots in the graph represent single protein quantifications from independent samples **(K)**. CNPase, 2′,3′-cyclic nucleotide 3′-phosphodiesterase; bFGF, basic fibroblast growth factor; DIV, day *in vitro*; EGF, epidermal growth factor; MBP, myelin basic protein; NG2, chondroitin sulfate proteoglycan, neural/glial antigen 2; NGF, nerve growth factor; p75^NTR^, low affinity nerve growth factor receptor; PDGF, platelet derived growth factor; PDGFαR, platelet derived growth factor alpha receptor; T3, triiodothyronine; TrkA, Tropomyosin receptor kinase A, TrkB: Tropomyosin receptor kinase B, TrkC: Tropomyosin receptor kinase C.

Fetal NSCs were isolated from E.13.5 forebrain as already described in a detailed methodological publication, including the culture characterization, the OGD, and the high content screening-based analysis of cell death and differentiation (Baldassarro, 2021; Figure 1A). In brief, tissues were incubated in non-enzymatic dissociation buffer (Sigma-Aldrich, Saint Louis, MO, USA) at 37°C for 15 min, then mechanically dissociated by pipetting. Cells were resuspended in serum-free medium (DMEM/F12 GlutaMAX; 8 mmol/L HEPES; 100 U/100 μg Penicillin/Streptomycin; 1 × B27; 1 × N2; 20 ng/mL bFGF; 20 ng/mL EGF; Thermo Fisher Scientific, Waltham, MA, USA) and plated in suspension, at a density of 10 cells/μl in flasks (Nunc, Roskilde, DK) kept in vertical to avoid cell adhesion. Half medium was changed every 3 days, centrifuging the cell suspension at 300 × g for 5 min and gently resuspending the cellular pellet in fresh medium. Neurospheres were allowed to proliferate until they attained a diameter of about 100 μm.

To obtain the oligospheres, primary neurospheres were centrifuged at 300 × g for 5 min. The pellet was mechanically dissociated by pipetting, and cells were counted and plated again at a density of 10 cells/μl in OPC medium (DMEM/F12 GlutaMAX; 8 mmol/L HEPES; 100 U/100 μg Penicillin/Streptomycin; 1 × B27; 1 × N2; 20 ng/mL bFGF; 20 ng/mL PDGF; Thermo Fisher Scientific, Waltham, MA, USA). The oligospheres were centrifuged and the pellet mechanically dissociated to obtain a single cell suspension. Following cell count, cells were plated at a density of 3,000 cells/cm^2^ on poly-D,L-ornithine (50 μg/ml)/laminin (5 μg/ml; Sigma-Aldrich) coating, in OPC medium.

To induce oligodendrocyte differentiation and maturation, the OPC medium was replaced with the oligodendrocyte differentiation medium (DMEM/F12 GlutaMAX; 8 mmol/L HEPES; 100 U/100 μg Penicillin/Streptomycin; 1 × B27; 1 × N2; 50 nM T3; 10 ng/ml CNTF; 1 × *N*-acetyl-L-cysteine – NAC; Thermo Fisher Scientific, Waltham, MA, USA) following 3 DIVs.

**FIGURE 2:**
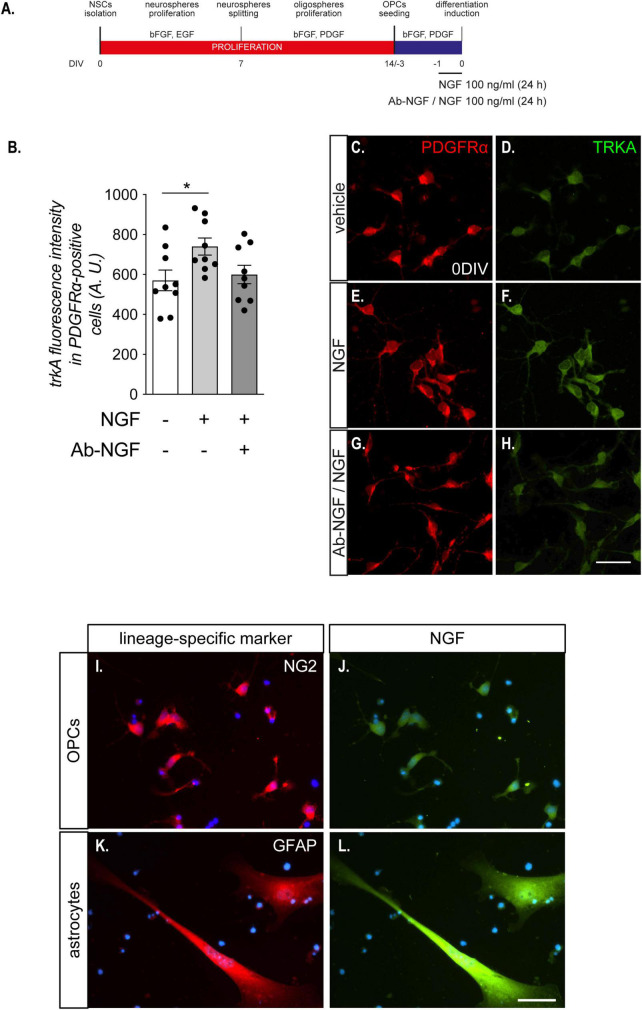
OPCs are sensitive to NGF treatment. **(A)** NSC-derived mixed OPC/astrocyte cultures were exposed to NGF (100 ng/ml) for 24 h prior to T3-mediated differentiation induction. In an independent experimental group, a 1 h pre-incubation of the solution containing NGF was performed with the specific anti-NGF antibody (Ab-NGF), before adding the solution in the culture medium. **(B)** Graph shows the TRKA immunofluorescence quantification in PDGFαR-positive cells in cultures untreated (with bar, *n* = 8), treated (light gray bar, *n* = 9) with NGF, or treated with NGF after the neutralization with the Ab-NGF (dark gray bar, *n* = 9). **(C–F)**. Representative images of OPCs (PDGFαR-positive cells, panels **C,E,G**), double positive for TRKA immunostaining **(D,F,H)**, treated with vehicle **(C,D)**, NGF **(E,F)**, or NGF neutralized by Ab-NGF **(G–H)**. Scale bar: 20 μm. **(I–L)** Representative images of NG2-NGF **(I–J)** or GFAP-NGF **(K–L)** double stained NSCs-derived OPC cultures at DIV 0. Scale bar: 10 μm. Bars represent mean ± SEM and single dots represent single images quantification, obtained from three independent experiments. Statistical analysis: Student’s *t*-test. Asterisks represent the differences between groups indicated by an horizontal line (*p < 0.05). A.U., arbitrary units; bFGF, basic fibroblast growth factor; DIV, day *in vitro*; EGF, epidermal growth factor; NGF, nerve growth factor; PDGF, platelet derived growth factor; PDGFαR, platelet derived growth factor alpha receptor; T3, triiodothyronine; TRKA, high affinity nerve growth factor receptor.

To characterize the responsiveness of OPCs to NGF in the mixed cultures, in a set of experiments we treated cultures with vehicle or NGF (100 ng/ml) at DIV 0 for 24 h (Figure 2A).

### 2.2. Primary astrocyte cultures

Primary astrocytes were isolated from single 7-day-old mice using a standard protocol (Baldassarro et al., 2017b), and cultured in DMEM containing 15% fetal bovine serum (FBS, Thermo Fisher Scientific, Waltham, MA, USA), non-essential amino acid mixture (Sigma-Aldrich), pen/strep (Thermo Fisher Scientific, Waltham, MA, USA) and 2 mM Glutamine (Thermo Fisher Scientific, Waltham, MA, USA). Cultures were seeded in culture-treated flasks at a density of 125,000 cells/cm2 and maintained at 37°C 5% CO2. Cells were detached with trypsin (10 min, 37°C) and plated twice before use. Two days before the culture medium collection for NGF quantification and OPCs protection, the medium was replaced with OPC medium. One day after medium change, astrocytes were exposed to OGD for 3 h (as described in the Section “Oxygen-glucose deprivation exposure and NGF or astrocyte conditioned medium treatments”) and, after 24 h of reoxygenation/reperfusion, medium was collected.

### 2.3. NGF and anti-NGF antibody preparation

Murine β-NGF from adult male mouse submaxillary glands was purified in our laboratory as already described (Bocchini and Angeletti, 1969), with minor revisions. Male Crl:CD1(ICR) mice aged 6–8 months were obtained from Charles River Italia (Calco, Varese, Italy) and sacrificed by cervical translocation. The anti-NGF antibody was raised in goat and purified following an established procedure (Stoeckel and Thoenen, 1975; Stoeckel et al., 1976).

### 2.4. Antibody anti-NGF and TRKA antagonist treatment

To investigate the role of endogenous NGF production in OPC cultures, we exposed cells to antibody anti-NGF (Ab-NGF; 10 μg/ml) or the TRKA antagonist GW-441756 (0.1, 1, or 10 μM; Tocris Bioscience, Bristol, UK), from DIV -1, i.e., 24 h prior to T3-mediated differentiation induction.

### 2.5. Oxygen-glucose deprivation exposure and NGF or astrocyte conditioned medium treatments

Oxygen-glucose deprivation (OGD) was performed using an air-tight hypoxia chamber (Billups-Rothenberg Inc., Del Mar., CA) saturated with 95% N_2_ and 5% CO_2_ as already described (Baldassarro et al., 2018, 2019b). In brief, glucose deprivation was achieved using a glucose-free medium (DMEM no glucose, Cat. A1443001; Thermo Fisher Scientific, Waltham, MA, USA) while oxygen was removed by flushing the hypoxia chamber with N_2_-CO_2_ mixture (95% – 5%) for 6–8 min at 25 l/min. These OGD conditions were maintained for 3 h, after which plates were re-oxygenated in the old glucose-containing medium in the incubator.

Cells were exposed to NGF or astrocytes conditioned medium (ACM) both in normoxia and OGD conditions, beginning 1 h before OGD and continuing until the end of differentiation.

In another set of experiments, cells were pre-treated for 1 h before OGD exposure with NGF (100 ng/ml) alone or with GW-441756 (1 μM). Drug treatment was performed also during OGD exposure, after which cells were fixed for AKT/pAKT staining.

### 2.6. Immunocytochemistry

Indirect immunofluorescence was used to identify OPCs (NG2 or PDGFαR-positive cells) and mature (CNPase-positive cells) and myelinating (MBP-positive cells) oligodendrocytes. Cultures were fixed in 4% of cold paraformaldehyde for 15 min and all primary and secondary antibodies used in the present study are listed in Table 1. For AKT/pAKT identification, during fixation a preotease/phosphatase inhibitor cocktail (PMSF 1 mM, sodium floride 10 mM, sodium orthovanadate 1 mM) was added. Cells were also incubated with the nuclear dye Hoechst 33258 (1 μg/mL in PBS, 0.3% Triton-X 100) to identify the nuclei.

**TABLE 1:** List of primary and secondary antibodies used in the immunocytochemistry reactions.

Antibody (company)	Species	Dilution
Anti-TRKA (abcam)	rabbit	1:250
Anti-p75^NTR^ (promega)	rabbit	1:250
Anti-NG2 (millipore)	rabbit	1:350
Anti-PDGFαR (santa cruz biotechnology)	mouse	1:300
Anti-CNPase (millipore)	mouse	1:250
Anti-MBP (dako)	rabbit	1:250
Anti-AKT (pan) (cell signaling technology)	rabbit	1:400
Anti-phospo-AKT (Ser473) (cell signaling technology)	rabbit	1:200
Alexa fluor 488-conjugated anti mouse (invitrogen)	donkey	1: 500
Alexa fluor 568-conjugated anti mouse (molecular probes)	goat	1:500
RRX-conjugated anti rabbit (jackson immunoresearch)	donkey	1:500
Alexa fluor 488-conjugated anti rabbit (molecular probes)	donkey	1:500

CNPase, 2′, 3′-cyclic nucleotide 3′-phosphodiesterase; MBP, myelin basic protein; NG2, chondroitin sulphate proteoglycan, neural/glial antigen 2; p75^NTR^, p75 neurotrophine receptor; PDGFαR, platelet derived growth factor alpha receptor; TRKA, tropomyosin receptor kinase A.

For each reaction a control group stained only with secondary antibodies was included to check for specificity.

### 2.7. Epifluorescence microscopy, confocal imaging, and IMARIS software elaboration

Fluorescence microscopy observations and photography were performed using a Nikon Eclipse E600 (Nikon, Tokyo, Japan) microscope equipped with the digital CCD camera Q Imaging Retiga-2000RV (Q Imaging, Surrey, BC, CA) and Nis-Elements AR 4.3 software.

For AKT/pAKT analysis mounted coverslips were scanned with a Nikon Ti-E fluorescence microscope coupled to an A1R confocal system (Nikon, Tokyo, Japan). A diode laser system with 405 wavelength output and an air-cooled Argon-Iron laser system with 488 wavelength output were used. Images were acquired with a 40 × objective using Nis-Element AR 4.3 software. All the *z*-stacks were collected in compliance with optical section separation (*z*-inteval) values suggested by the software (1 μm).

The *z*-stacks were elaborated and analyzed by IMARIS software (v. 7.7.2; Andor Technology, Belfast, UK). The reconstruction of nuclear isosurfaces was based on the Hoechst 33258 nuclear staining fluorescence and OPCs nuclei were selected based on nuclear size and cell morphology. In fact, OPCs show smaller nuclei than astrocytes and small and round cell body with short ramifications totally different from astrocytes which show fibroblasts-like flat cell body. Fluorescence intensity of AKT or pAKT staining, with the automatic application of background removal, was measured only inside the reconstructed volume of the nuclear isosurfaces by the dedicated software algorithm (mean intensity).

### 2.8. High content screening

For High content screening (HCS) analysis, cells were grown in 96 flat-bottom well HCS plates (Nunc). Analysis of condensed nuclei, cell number and lineage/differentiation markers were performed with Cell Insight CX5 High Content Screening (HCS, Thermo Fisher Scientific, Waltham, MA, USA), using the *Compartmental analysis* BioApplication and a 10x objective (UPlanFl N, 10 × /0.30, FN26.5, Olympus, Segrate, MI, Italy). Based on nuclear staining, the software is able to recognize nuclei and identify the high intensity/small-sized condensed nuclei, measuring the number of normal nuclei per well (viable cells) and the percentage of condensed nuclei (cell death). Moreover, based on the nuclei identification, the software is able to detect the presence of the marker-specific staining in the cell body, calculating the percentage of immunoreactive cells. Lineage/differentiation markers analysis was performed on cells showing intact nuclei only, excluding condensed nuclei. The HCS system allows the analysis of the whole culture, avoiding the bias inherent in choosing random fields. An average number of 30,000 cells/wells were included in the analysis.

### 2.9. RNA isolation and reverse transcription

Total RNA isolation was performed using the RNeasy Plus Micro kit (Qiagen, Hilden, DE) following manufacturer’s instructions. Total RNA was eluted in RNase-free water and concentration estimated using Nanodrop 2000 spectrophotometer (Thermo Fisher Scientific, Waltham, MA, USA). First-strand cDNAs were obtained using the iScript™ cDNA Synthesis Kit (Bio-Rad, CA, USA), incubating at 42°C for 30 min. An RNA sample with no reverse transcriptase enzyme in the reaction mix was processed as a no-reverse transcription control sample.

### 2.10. Semi-quantitative real-time PCR

Semi-quantitative real-time PCR was performed using the CFX96 real-time PCR system (Bio-Rad). The reactions were performed in a final volume of 20 μl consisting of 1 × SYBR Green qPCR master mix (Bio-Rad) and 0.4 μM forward and reverse primers. To avoid possible contamination of genomic DNA in isolated RNA, the sample with no-reverse transcriptase enzyme was processed in parallel with the others and tested using real-time PCR for the housekeeping gene. All primers used were designed using Primer Blast software (NCBI, MD, USA) and synthesized by IDT (Coralville, IA, USA). The primer sequences are listed in Table 2. GAPDH was used as housekeeping gene to normalize the amount of reverse-transcribed RNA used for PCR. The thermal profile of the PCR reactions consisted of an initial denaturation step (95°C, 2 min) and 40 cycles of amplification (95°C for 15 s and 60°C for 60 s). At the end of the amplification cycles, the melting curve of the amplified products was performed according to the following temperature/time scheme: heating from 55°C to 95°C with a temperature increase of 0.5°C/s. The 2^−(ΔΔ*Ct*)^ method was used for the calculation of gene expression.

**TABLE 2:** List of the primer sequences.

GeneBank	RefSeqs	FW (5′–3′)	REV (5′–3′)
*Ngf*	NM_001112698.1; NM_013609.2	ACCTCTTCGGACACTCTG	CGTGGCTGTGGTCTTATCTC
*TrkA (Ntrk1)*	NM_001033124.1	TGCCTTCCGTTTCACCCCTCG	CCCTTCCTGCTCCCAACGCT
*TrKB (Ntrk2)*	NM_001025074.2; NM_008745.3; NM_001282961.1	TCCAGCCCCGACACTCAGGAT	CCAGTACAAGGTGGGGAGTGGG
*TrKC (Ntrk3)*	NM_008746.5; NM_182809.2	GGACATGGAGCTCTACACGG	TGCTCCAGTCTCAATTCCCG
*p75^NTR^ (Ngfr)*	NM_033217.3	AGTGGCATCTCTGTGGAC	CTACCTCCTCACGCTTGG
*Gapdh*	NM_001289726.1/NM_008084.3	GGCAAGTTCAATGGCACAGTCAAG	ACATACTCAGCACCAGCATCACC

Gapdh, glyceraldehyde 3-phosphate dehydrogenase; Ngf, nerve growth factor; Ngfr, nerve growth factor receptor; p75^NTR^, p75 neurotrophine receptor; Ntrk, neurotrophic tyrosine kinase; TrkA, tropomyosin receptor kinase A; TrkB, tropomyosin receptor kinase B; TrkC, tropomyosin receptor kinase C.

### 2.11. NGF protein quantification in culture medium

Nerve growth factor protein levels were evaluated using two immunoassays based on xMAP technology: a species-specific kit for mouse NGF (R&D Systems, Mouse Magnetic Luminex Assay, cat. LXSAMSM-1) and a high-sensitivity kit for human NGF detection (Merck-Millipore, Adipokine Magnetic Bead Panel 2, cat. HADK2MAG-61K).

To the best of our knowledge, the R&D systems kit is the only commercially available kit for mouse NGF detection but, in our experience, it was unable to detect and quantify the protein in the cell culture medium, prompting us to switch to the highly sensitive Merck-Millipore kit.

Following the manufacturer’s instructions, samples were first incubated with antibody-immobilized bead solution (capture antibody), followed by detector antibody incubation and finally with streptavidin-phycoerythrin buffer. Fluorescence intensity was detected by the MAGPIX Luminex instrument.

To obtain mouse NGF concentrations, fluorescence values were interpolated on a standard curve produced by serial dilutions of mouse NGF protein. For the R&D Systems kit, the mouse NGF standard curve ranged from 1.15 to 840 pg/ml (7 standards, dilution factor = 3), whereas for the Merck-Millipore kit it ranged from 0.64 to 10,000 pg/ml (7 standards, dilution factor = 5). The softwares xPONENT v 3.1 (Luminex xMAP technologies) and Milliplex Analyst v 3.5.5 (Merck-Millipore) respectively, were used for analysis. Results were accepted if the *r*^2^ of the standard curve was ≥ 0.95.

The Merck-Millipore kit includes a monoclonal capture anti-NGF antibody that recognizes the Ala 46-Asn 62 epitope, conserved in the mouse protein (Paoletti et al., 2015). The highly accurate and precise standard curve obtained using defined concentrations of mouse NGF diluted in culture medium was used for the validation of the detection kit and for the quantification of the protein in the samples (*r*^2^ = 1; mean accuracy = 101.7%).

### 2.12. Statistical analysis

Data is reported as mean ± SEM. Prism software (GraphPad, Boston, MA, USA) was used for statistical analyses and graph generation. Student’s *t*-test, one-way ANOVA followed by Dunnett’s post-test, Kruskal-Wallis test followed by Dunn’s post-test or two-way ANOVA followed by Sidak’s post-test were used to analyze the data. Results were considered significant when the probability of their occurrence as a result of chance alone was less than 5% (*p* < 0.05). All the experiments were performed in at least three independents replicates for each experimental group. Number of replicates are showed as single dots in the graphs and specified in the figure legends.

## 3. Results

### 3.1. NGF and NGF receptors are dynamically expressed throughout differentiation and maturation of OPCs derived from NSCs

By using specific growth factors (bFGF/EGF), it is possible to induce proliferation of multipotent stem cells derived from the fetal brain and push them to the OL lineage (bFGF/PDGF-AA) (Figure 1A). By plating OPCs on laminin-coated wells, and in the presence of T3, cells switch from a proliferative state to differentiation, finally maturing as OLs (Figures 1B–D). This differentiated culture also contained astrocytes (30–40%) throughout the entire culture process (Baldassarro et al., 2019a).

We first analyzed the mRNA expression of the genes encoding for the neurotrophin receptors at all differentiation stages, from proliferating neurospheres (Neuros) and oligospheres (Oligos) to differentiated OLs.: the high affinity NGF recptor, *TrkA*; the BDNF receptor *TrkB*, the NT3 receptor *TrkC*; and the low-affinity NGF receptor *p75^NTR^*.

The gene expression analysis shows a significant regulation dependent on the culture time of all the neurotrophin receptors except for *TrkB* (Kruskal-Wallis test; *TrkA*, *p* = 0.0449; *TrkB*, *p* = 0.0560; *TrkC*, *p* = 0.0132; *p75^NTR^*, *p* = 0.0088; Figures 1E–H. However, TrkA is the only receptor showing an expression peak at the differentiation induction (1 DIV). The *TrkA* expression peak corresponded also to the lowest expression level of *p75^NTR^*, suggesting a dynamic and opposite action of the two receptors.

The NGF receptors regulation also parallels to *Ngf* gene expression in the astrocyte/OPC mixed culture (Kruskal-Wallis test, *p* = 0.0054; Figure 1L), with a boost in the expression corresponding to the expression peak of *TrkA* gene.

As specified previously, both OPCs/OLs (60–70%) and astrocytes (30–40%) are present in the cell system, as showed in the culture composition characterization (Figure 1J) and as detailed explained in previous publications (Baldassarro, 2021). In fact, we also quantified the NGF protein released by the cells in the culture medium, finding an increase of the secreted protein by time, reaching a peak at the end of the differentiation stage (Kruskal-Wallis test, *p* < 0.0002; Figure 1K). However, the dynamic expression of NGF receptors, linked also to the T3-induced differentiation, and the increase of *Ngf* expression and secretion, suggested a direct involvement of NGF in the differentiation process of the OPCs. We thus focused on this neurotrophin for the following experiments and, on the mixed cultures, we performed double staining immunocytochemistry to investigate if OPCs express NGF receptors and who is the main producer of NGF in the mixed culture. We find that cells expressing the OPC marker PDGFαR are also positive for TRKA and p75*^NTR^* already at 0 DIV (Figures 1L–O).

Notably, TRKA expression in PDGFRα-positive OPCs was modified by the presence of the NGF in the culture medium (one-way ANOVA, *F*_(2_,_24)_ = 3.680; *p* = 0.0404). TRKA protein level, in fact, was upregulated in OPCs by 24 h of NGF exposure (100 ng/ml) (Tukey’s post-test, *p* = 0.0458; Figure 2B), while this regulation was totally blocked by the pre-incubation of NGF with the anti NGF antibody, demonstrating that the observed difference was directly mediated by the growth factor. Interestingly, we did not observe any effect on p75*^NTR^* immunostaining (*data not shown*). Representative images of TRKA immunostaining in PDGFRα-positive cells exposed or not to NGF are included in the panel (Figure 2C–H).

Using the anti-NGF antibody we also found that OPCs (NG2-positive cells) are poorly labeled for the NGF expression, while astrocytes (GFAP-positive cells) are the main cell type producing NGF (Figures 2I–L).

### 3.2. Endogenous NGF production promotes proper OPC differentiation

**FIGURE 3:**
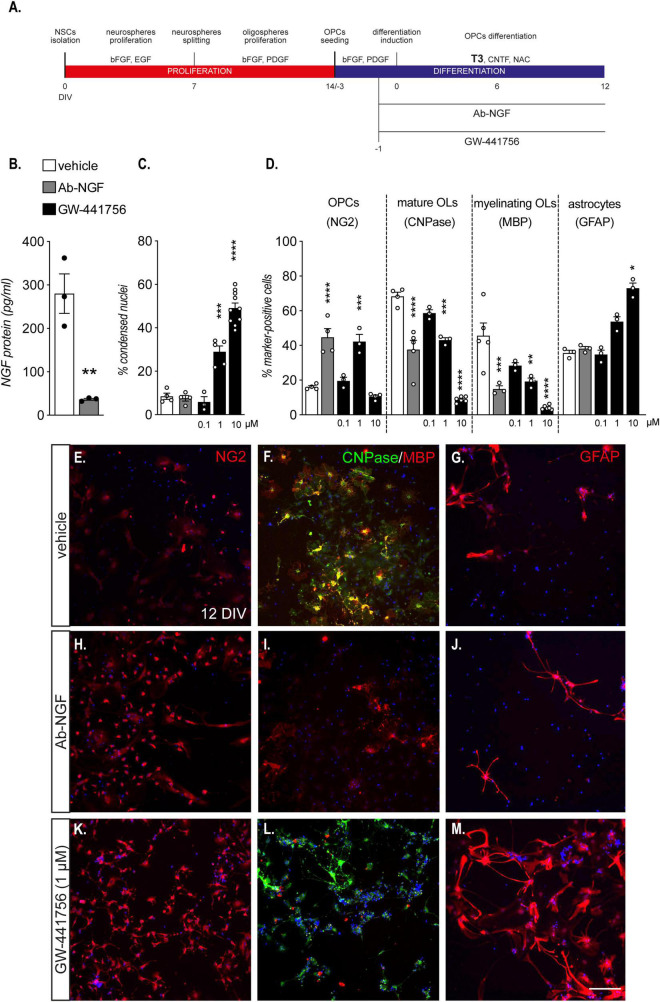
Endogenous NGF is essential for OPCs differentiation. **(A)** NSC-derived mixed OPC/astrocyte cultures were exposed to an antibody anti-NGF (Ab-NGF) or GW-441756 from 24 h prior to T3 exposure (–1 DIV) to the end of the differentiation (12 DIV). **(B)** Graph shows the absolute NGF protein quantification (pg/ml) at the end of the differentiation phase (12 DIV) in cultures treated with vehicle or Ab-NGF (*n* = 3). **(C)**: Graph shows the percentage of cell death expressed as percentage of condensed nuclei at the end of differentiation phase (12 DIV) in cells treated with vehicle (*n* = 4), Ab-NGF (*n* = 4) or different concentrations of GW-441756 (0.1 μM, *n* = 3; 1 μM, *n* = 5; 10 μM, *n* = 10). **(D)** Graph shows the percentage of marker-specific cells for OPCs (NG2), mature OLs (CNPase) and myelinating OLs (MBP), at the end of the differentiation phase (12 DIV) in cells treated with vehicle (NG2, *n* = 4; CNPase, *n* = 4; MBP, *n* = 5; GFAP, *n* = 3), Ab-NGF (NG2, *n* = 4; CNPase, *n* = 4; MBP, *n* = 3; GFAP, *n* = 3) or different concentrations of GW-441756 (0.1 μM, *n* = 3; 1 μM, *n* = 3; 10 μM, NG2, *n* = 4; CNPase, *n* = 6; MBP, *n* = 3). E – M: Representative images of cells stained for NG2 **(E,H,K)**, CNPase/MBP **(F,I,L)** and GFAP **(G,J,M)** of cultures treated with vehicle **(E–G)**, Ab-NGF **(H–J)** or GW-441756 1 μM **(K–M)**. Scale bar **(M)**: 50 μm. Bars represent mean ± SEM and single dots in the graphs represent single protein quantifications from independent samples **(B)** or independent experiments analyzed by high content screening **(C,D)**. Statistical analysis: Student’s *t*-test **(B)**, One-way ANOVA followed by Dunnet’s post-test **(C,D)** or Kruskal-Wallis test followed by Dunn’s post-test (panel **D**, GFAP). Asterisks represent the differences between cultures treated with vehicle or Ab-NGF (* *p* < 0.05; ** *p* < 0.01; *** *p* < 0.001; **** *p* < 0.0001). Ab-NGF, antibody anti-nerve growth factor; CNPase, 2′,3′-cyclic nucleotide 3′-phosphodiesterase; bFGF, basic fibroblast growth factor; DIV, day *in vitro*; EGF, epidermal growth factor; MBP, myelin basic protein; NG2, chondroitin sulphate proteoglycan, neural/glial antigen 2; NGF, nerve growth factor; OLs, oligodendrocytes; OPCs, oligodendrocyte precursor cells; PDGF, platelet derived growth factor; T3, triiodothyronine.

The developmental regulation of NGF synthesis in an OPC-enriched culture system suggests that the NGF pathway is directly or indirectly implicated in OPC differentiation. To confirm this hypothesis, we performed NGF neutralization experiments by treating the culture with NGF antibody or GW-441756, a potent and selective TRKA antagonist (Wood et al., 2004), starting 24 h prior to T3-induced differentiation and continuing until the end of the differentiation phase (Figure 3A).

We initially quantified the NGF protein in the medium of cells treated and untreated with anti-NGF antibody, to confirm the efficacy of the antibody-mediated neutralization protocol (Figure 3B). The antibody treatment strongly reduced the protein level in the medium (Student’s *t*-test, *p* = 0.0059), suggesting that the binding site recognized by the diagnostic anti-NGF antibody was already occupied by the neutralizing antibody.

The NGF neutralization by the specific antibody and the lower dose of TRKA antagonist (0.1 μM) does not affect cell viability, while highest concentrations of GW-441756 produced an increase in the percentage of condensed nuclei [One-way ANOVA, *F*_(4_, _21)_ = 67.43, *p* < 0.0001; 1 μM, *p* = 0.0001; 10 μM, *p* < 0.0001; Figure 3C]. Cell death induction by the GW-441756 molecular tool was also validated using LDH assay (Supplementary Figure 1).

The reduction of both NGF availability in the culture medium and the TRKA activity are translated to a reduction in OPC differentiation (Figure 3D): indeed the percentage of OPCs (NG2-positive cells) at the end of the differentiation period increased with the antibody anti-NGF and the GW-441756 at 1 μM [One-Way ANOVA, *F*_(4_, _13)_ = 26.84, *p* < 0.0001; Ab-NGF, *p* < 0.0001; GW-441756 1 μM, *p* = 0.0002], while the percentage of mature (CNPase-positive cells) and myelinating OPCs (MBP-positive cells) is strongly reduced with Ab-NGF treatment and GW-441756 higher doses exposure (One-Way ANOVA; CNPase, *F*_(4_,_16)_ = 59.20, *p* < 0.0001; Ab-NGF, *p* < 0.0001; GW-441756 1 μM, *p* = 0.0004; 10 μM, *p* < 0.0001; MBP, *F*_(4_,_15)_ = 16.26, *p* < 0.0001; Ab-NGF, *p* = 0.0009; GW-441756 1 μM, *p* = 0.0033; 10 μM, *p* < 0.0001). In parallel with the effect on cell viability, GW-441756 exposure at cytotoxic concentration correspond to an increase in GFAP-positive cell percentage (Kruskal-Wallis test, *p* = 0.0015; 10 μM, *p* = 0.0325) suggesting a specific OL lineage cell death. Representative images of HCS captured fields of anti-NGF and GW-441756 (1 μM) treated cultures are shown (Figure 3E–M).

### 3.3. NGF treatment protects OPCs from OGD-mediated toxicity and differentiation impairment

Previously reported results showed a physiological role of the NGF produced by mixed OPC/astrocyte cultures, revealing that NGF availability is necessary for OPC differentiation.

We then proceeded to investigate whether NGF also plays a role in OPC survival and differentiation in pathological conditions. Given that OPCs are highly vulnerable to hypoxic/ischemic conditions (Baldassarro et al., 2019b), we studied the response of this cell system to OGD in the presence of NGF.

**FIGURE 4:**
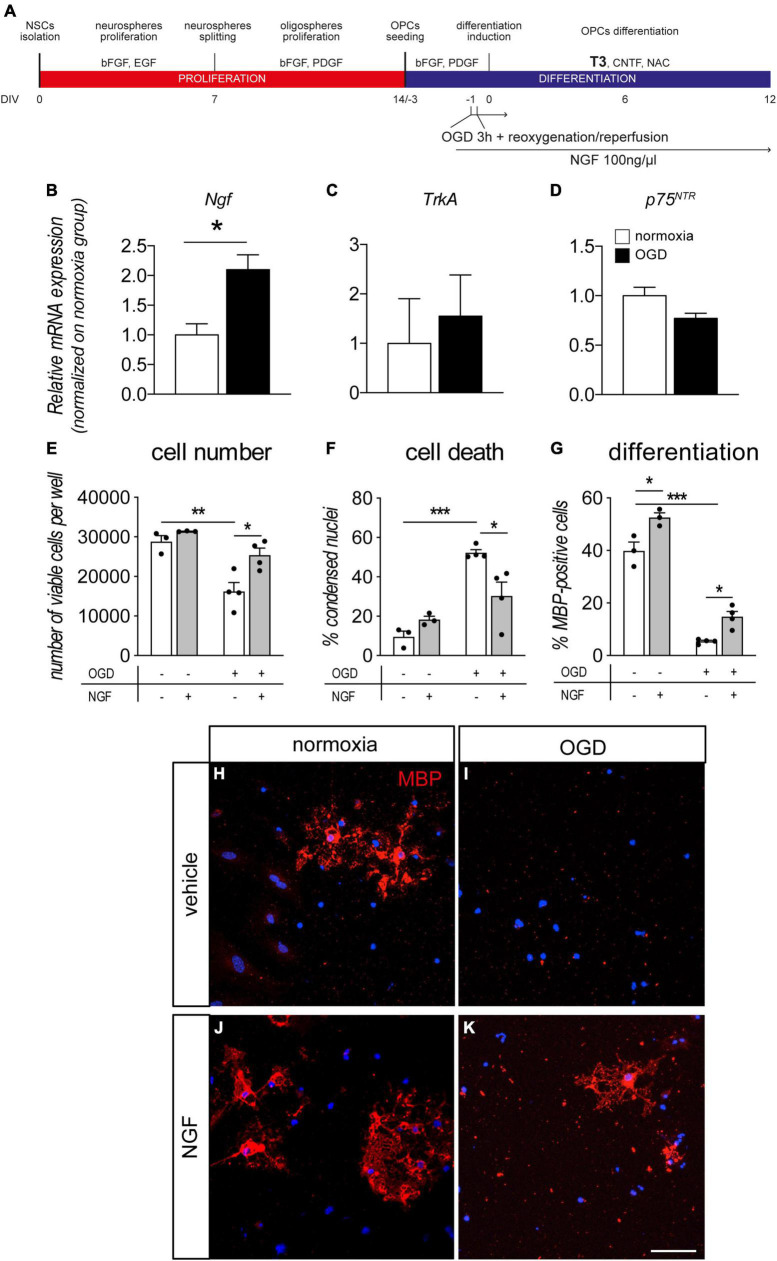
NGF boosts OPC differentiation and protects from OGD. **(A)** NSC-derived mixed OPC/astrocyte cultures were exposed to OGD 24 h prior to T3-mediated differentiation induction (–1 DIV). Cells were treated with vehicle or NGF (100 ng/ml) beginning 1 h prior to OGD. Cultures were analyzed for gene expression 24 h after OGD exposure (0 DIV) and for cell number/cell death and differentiation at the end of the differentiation phase (12 DIV). **(B–D)** Graphs show relative mRNA expression of *NGF* **(B)**, *TrkA* **(C)** and *p75^NTR^* **(D)** genes, following 3 h of OGD and 24 h of reoxygenation/reperfusion, in culture exposed and unexposed to OGD and normalized on normoxia-exposed groups; *n* = 3 for all the presented groups. **(E–G)** Graphs show cell number **(E)**, percentage of condensed nuclei (cell death, panel **F**) and percentage of MBP-positive cells (cell differentiation, panel **G**), in cultures exposed to normoxia (*n* = 3) or OGD (*n* = 4) and treated or untreated with NGF (100 ng/ml) (normoxia, *n* = 3; OGD, *n* = 4), at the end of the differentiation phase (12 DIV). **(H–K)** Representative images of MBP-positive cells (myelinating OLs) in cultures exposed to normoxia **(H,J)** or OGD **(I,K)** and treated with vehicle **(H,I)** or NGF **(J,K)**. Scale bar: 20 μm. Bars represent mean ± SEM and single dots in the graphs represent single independent experiments analyzed by high content screening **(E–G)**. Statistical analysis: Student’s *t*-test **(B–D)** or two-way ANOVA followed by Sidak’s post-test **(E–G)**. Asterisks represent the differences between indicated groups (*p < 0.05; **p < 0.01; ***p < 0.001). bFGF, basic fibroblast growth factor; DIV, day *in vitro*; EGF, epidermal growth factor; MBP, myelin basic protein; NGF, nerve growth factor; OGD, oxygen-glucose deprivation; OLs, oligodendrocytes; p75^NTR^, low affinity nerve growth factor receptor; PDGF, platelet derived growth factor; T3, triiodothyronine.

First, we analyzed the NGF gene expression in cells exposed or not to OGD, followed by 24 h of reoxygenation/reperfusion. We found that *Ngf* expression increased following OGD (Student’s *t*-test, *p* = 0.0219; Figure 4B), while *TrkA* and *p75^NTR^* mRNA expression levels were not affected (Figure 4C, D).

In another set of experiments, we pre-treated cultures with NGF before OGD or normoxia, finally analyzing the cell viability and OL maturation at the end of the differentiation stage (12 DIV, Figures 4E–G). As already described by our group, OGD exposure induced cell death in OL-lineage cells (Baldassarro et al., 2019b). NGF treatment protected the cell system by increasing the cell number (Figure 4E, two-way ANOVA, OGD *F*_(1–10)_ = 25.93, *p* = 0.0005, treatment F_(1–10)_ = 10.53, *p* = 0.0088); and decreasing the percentage of condensed nuclei (Figure 4F, two-way ANOVA, OGD *F*_(1–10)_ = 36.80, *p* = 0.0001, interaction *F*_(1–10)_ = 11.67, *p* = 0.0066). In particular, NGF treatment strongly reduced the OGD-induced cell death (cell number, *p* = 0.0189; percentage of condensed nuclei, *p* = 0.0232). The protective effect of the exogenous NGF administration in OGD condition was also validated using LDH assay (Supplementary Figure 1).

Moreover, the percentage of myelinating OLs in the differentiated cultures (MBP-positive cells) revealed that both OGD and NGF treatment affect culture composition (Figure 4G, two-way ANOVA, OGD *F*_(1–10)_ = 319.8, *p* < 0.0001; treatment *F*_(1–10)_ = 29.96, *p* = 0.0003). OGD exposure, in fact, leads to a strong decrease in the percentage of MBP-positive cells in cultures (Sidak post-test *p* < 0.0001), while NGF treatment is able to increase their percentage both in normoxia (*p* = 0.115) and OGD-exposed (*p* = 0.0311) cultures, confirming the positive effect of this growth factor in the differentiation/maturation process. Representative images of cultures exposed or not to OGD and to NGF treatments are included in the figure (Figures 4H–K).

### 3.4. Astrocytes conditioned medium exposure protects OPCs from OGD-mediated toxicity

**FIGURE 5:**
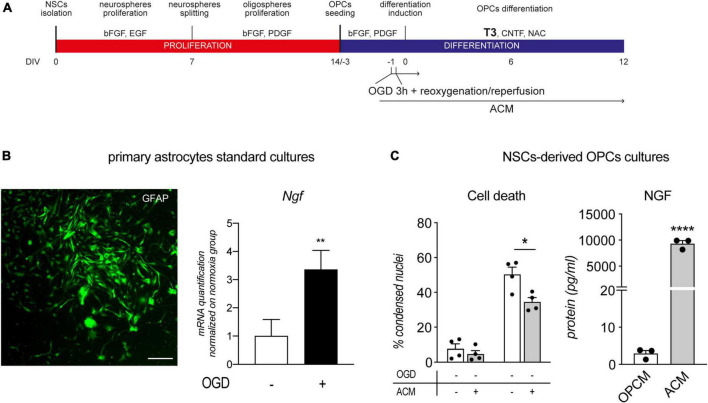
Astrocytes conditioned medium protects OPCs from OGD. **(A)** NSC-derived mixed OPC/astrocyte cultures were exposed to OGD 24 h prior to T3-mediated differentiation induction (–1 DIV) and from 1 h before OGD exposure, to ACM. **(B)** Representative picture of GFAP stained primary astrocytes culture and RNA quantification of NGF gene expression following 3 h of OGD and 24 h of reoxygenation/reperfusion, in culture exposed and unexposed to OGD and normalized on normoxia-exposed groups (*n* = 3 for both groups). Scale bar: 100 μM. **(C)** Graphs show the percentage of condensed nuclei in cultures exposed to normoxia or OGD and treated or not with ACM, at the end of the differentiation phase (12 DIV), *n* = 4 for all the presented groups; and the quantification of NGF protein in oligodendrocyte precursor cell medium (OPCM) and astrocyte conditioned medium (ACM), *n* = 3. Bars represent mean ± SEM and single dots in the graph represent single independent experiments analyzed by high content screening (panel **C**, cell death) or single protein quantification from independent well (panel **C**, NGF). Statistical analysis: Student’s *t*-test (*Ngf* gene expression, panel **B**; NGF protein quantification, panel **C**); Two-way ANOVA followed by Sidak’s post-test (Cell death, panel **C**). Asterisk represents the difference between indicated groups (*p < 0.05; **p < 0.01; ****p < 0.0001). ACM, astrocytes conditioned medium; bFGF, basic fibroblast growth factor; DIV, day *in vitro*; EGF, epidermal growth factor; MBP, myelin basic protein; NG2, chondroitin sulphate proteoglycan, neural/glial antigen 2; NGF, nerve growth factor; NSC, neural stem cell; OGD, oxygen-glucose deprivation; OPCM, oligodendrocyte precursor cell medium; PDGF, platelet derived growth factor; T3, triiodothyronine.

To confirm that astrocyte secretome is the possible source of NGF overcoming OPCs cell death induced by OGD, we prepared a culture of primary cortical astrocytes using the OPC culture medium, that were then exposed to OGD, and the conditioned medium was used to overcome OGD-induced OPCs cell death (Figure 5A).

After two passages and 1 week in culture, primary astrocytes are all positive for GFAP marker and OGD exposure of astrocytes cultures resulted in increase of NGF mRNA expression (Student’s *t*-test, *p* = 0.0063; Figure 5B). Since the immunocytochemistry experiments proved the production of NGF by astrocytes, we collected the medium after 48 h and quantified the NGF, resulting in an accumulation of NGF protein of 93.4 ± 21.52 pg/ml in 1 ml. The same medium was used on OPC cultures exposed or not to OGD, from 24 h before the differentiation phase (12 DIV). The ACM has no effect on the standard culture, while showed a reduction in the percentage of condensed nuclei in the OGD-exposed cells [Two-Way ANOVA; OGD, *F* (1,12) = 146.2, *p* < 0.0001; ACM, *F* (1,12) = 9.834, *p* = 0.0086; Sidak’s post-test, OGD-vehicle vs. OGD-ACM, *p* = 0.0172; Figure 5C, cell death]. The ACM is enriched by the astrocyte secreted factors in which NGF may be responsible for the protective effect. In fact, we quantified the NGF protein in the ACM, which resulted more than 1000 concentrated compared to the OPC medium at DIV 0 (Student’s *t*-test, *p* < 0.0001; Figure 5C, NGF).

### 3.5. NGF/TRKA action on OPCs is mediated by phosphorilized AKT translocation in the nucleus

**FIGURE 6:**
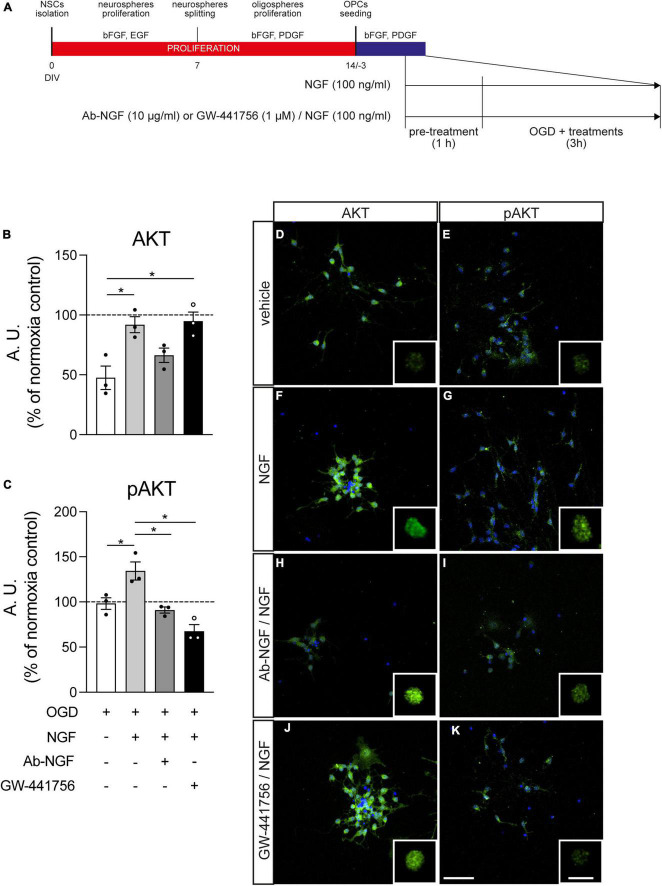
Effect of NGF treatment on Akt and pAkt presence in the nucleus. **(A)** NSC-derived mixed OPC/astrocyte cultures were pre-treated with vehicle or NGF (100 ng/ml) alone or in combination with GW-441756 (1 μM). Cultures were exposed to 3 h OGD and then fixed and stained for AKT or pAKT in combination with Hoechst 33258 nuclear staining. **(B,C)** Graphs show the quantification of AKT **(B)** or pAKT **(C)** fluorescence intensity inside the nucleus of cultures exposed to OGD and treated with vehicle (white bar), NGF (gray bar) or NGF with Ab-NGF (dark gray bar) or GW-441756 (black bar), normalized on the normoxia-vehicle treated group (horizontal dotted line); *n* = 3 for all the presented groups. **(D–K)** Representative pictures of OGD exposed cultures stained for AKT **(D,F,H,J)** or pAKT **(E,G,I,K)** and treated with vehicle **(D,E)**, NGF **(F,G)** or NGF and Ab-NGF **(H,I)** or GW-441756 **(J,K)**. Scale bar: 20 μm. For each panel the detail of the masked AKT/pAKT staining for the nuclear volume obtained by IMARIS software is included. Scale bar: 10 μm. Bars represent mean ± SEM and single dots in the graphs show a single independent replicate generated by the average value of five different pictures. Statistical analysis: Kruskal-Wallis test followed by Dunn’s post-test. Asterisks represent the difference between indicated groups (*p < 0.05). bFGF, basic fibroblast growth factor; DIV, day *in vitro*; EGF, epidermal growth factor; NGF, nerve growth factor; NSC, neural stem cell; OGD, oxygen-glucose deprivation; T3, triiodothyronine.

We described that NGF produced by astrocytes or added to the culture medium is able to protect OPCs from OGD-induced cell death and that blocking TRKA impairs OPCs survival and differentiation. Since AKT phosphorylation and nuclear translocation is a well-described mechanism of NGF action through TRKA receptor in other cell types (Xuan Nguyen et al., 2006), in another set of experiments we quantified the presence of AKT and pAKT in the OPCs nucleus by an immunocytochemistry-based assay using validated antibodies (Molgaard et al., 2016). We used 1 h of pre-treatment with NGF with or without Ab-NGF or GW-441756 before the OGD exposure, to ensure the TRKA block before the noxious stimulus, and we fixed the cells 3 h after OGD to identify the early response of AKT/pAKT (Figure 6A). Images were taken by a laser scan confocal microscope to acquire *z*-stacks and 3D images that were then analyzed by IMARIS software. Immunocytochemistry technique was preferred to other protein quantification methodology to identify the cell type responsible for AKT/pAKT changes, because the *in vitro* system contains both OPCs (at this differentiation stage: 70%) and astrocytes (at this differentiation stage: 30%), as already described (Baldassarro et al., 2017a; Baldassarro, 2021). Nuclei isosurfaces were reconstructed by IMARIS software based on the fluorescence of the Hoechst nuclear staining, and only OPC nuclei were selected for the analysis, based on the smaller size and the cell morphology (see white nuclei isosurfaces in Supplementary Figure 2). NG2- and GFAP-stained cultures were, in fact, previously analyzed and visualized for the nuclear size and cell morphology. Using IMARIS software we automatically selected the nuclei depending on the surrounding positivity to lineage-specific markers and we used isosurface reconstruction based on Hoechst staining to generate and measure the OPC and astrocyte nuclear volume (Supplementary Figure 3). AKT/pAKT expression was measured as mean fluorescence intensity inside the nucleus, using the dedicated IMARIS software algorithm (mean intensity in surface tool).

Nerve growth factor treatment increases the presence of AKT inside the nucleus, measured as fluorescence intensity (Kruskal-Wallis test, *p* = 0.0032; Dunn’s post-test, OGD vs. OGD-NGF, *p* = 0.0478) and the simultaneous block of TRKA by the specific inhibitor GW-441756 does not modify this effect (OGD vs. OGD-NGF-GW-441756, *p* = 0.0276). On the other hand, the neutralization of NGF through the specific antibody (Ab-NGF) blocks the effect of the growth factor, since no differences were detected between the OGD and the OGD-NGF-Ab-NGF groups (Figure 6B). NGF induces also an increase of the phosphorylated AKT (fluorescence intensity of pAKT staining) in the nucleus (Kruskal-Wallis test, *p* = 0.0006; Dunn’s post-test, OGD vs. OGD-NGF, *p* = 0.0386), totally inhibited by both the Ab-NGF (OGD-NGF vs. OGD-Ab-NGF, *p* = 0.0148) and the TRKA antagonist (OGD-NGF vs. OGD-NGF-GW-441756, *p* = 0.0134; Figure 6C). The treatment with only GW-441756 did not affected the AKT and pAKT levels (*data not shown*).

Figure 6 also includes representative images of cultures marked for AKT (D, F, H, J) and pAKT (E, G, I, K) in cells exposed to OGD and treated with vehicle (D, E), NGF (F, G), Ab-NGF/NGF (H, I), and GW-441756/NGF (J, K), with the detail of the masked AKT/pAKT channel inside the nuclear volume for each group.

## 4. Discussion

Nerve growth factor was discovered due to its roles in nervous system development and maturation, but it is also directly involved in physiological processes in the adult CNS, and exerts important functions in response to different injuries (Rocco et al., 2018). In the CNS, its main and best recognized target is the neuron and neuronal axon in NGF-sensitive populations, such as cholinergic neurons (Claudio Cuello et al., 2019). However, other cellular targets in the nervous system and in peripheral tissues are emerging (Aloe et al., 2015). In particular, a role of NGF in OPC/OL biology has been hypothesized, due in part to the fundamental role of these cells as a functional part of axonal structure, communication mechanisms and metabolism (Amaral et al., 2016), but data which emerged during the 1990s and early 2000s regarding the action of this neurotrophin on OPCs and mature OLs has generated a certain degree of confusion.

Based on our hypothesis that the different cell systems used for these studies might be partly responsible for this confusion, we decided to use a spontaneous mixed OPC/astrocyte *in vitro* system derived from fetal NSCs to highlight a possible role of NGF in OPC biology. This cell system has the limitation to not include any axonal myelination however, it allows OL maturation in approximately 30 days and reflects the full *in vivo* T3-dependent differentiation of OLs from NSCs (Baldassarro et al., 2019a,b; Sock and Wegner, 2019). Moreover, the constant presence of astrocytes in the mixed culture better resembles the physiological *in vivo* microenvironment.

Here we show a dynamic expression of all the neurotrophin receptors (*TrkA*, *TrkB*, *TrkC*, and *p75^NTR^*) during OPC generation and differentiation, suggesting a role of NGF, BDNF, and NT-3 in the OPC differentiation process. However, only the NGF receptors *TrkA* and *p75^NTR^*, show a regulation dependent on T3-induced differentiation induction, with a clear peak of *TrkA* expression 24 h following T3 exposure. This regulation is also directly linked to a parallel increase in *Ngf* expression and NGF protein secretion.

The data on neurotrophins receptors expression are in line with emerging roles of these molecules in in OPC differentiation and maturation, which are still poorly described, as recently proved for BDNF (Khani-Habibabadi et al., 2022). In the first part of this study, in fact, we demonstrated that mixed OPC/astrocyte cultures produce NGF and respond to NGF during OPC differentiation and OL maturation; that NGF receptors are dynamically expressed throughout the NSC lineage and OPC differentiation process, and that neutralization of endogenous NGF determines an impairment in OPC differentiation. Previously published data indicated that cultured adult human primary OLs seem to selectively express the *p75^NTR^* low-affinity neurotrophin receptor (Ladiwala et al., 1998), leading to the evidence that its activation by NGF treatments may drive cell death induction, both in primary rodent OL cultures (Casaccia-Bonnefil et al., 1996) and *in vivo* lesions (Brown et al., 2004). In primary OL cultures, this toxic effect seems to be absent from OPCs, restricted to mature OLs and reverted by TRKA activation (Casaccia-Bonnefil et al., 1999). Other studies using the same cell system have failed to confirm this result (Starkey et al., 2001), while other research groups have concluded that both OLs and Schwan cells, the myelinating cells in the peripheral nervous system, do not express *TrkA* when cultured in dorsal root ganglia neuron co-cultures, showing a specific pro-myelinating effect of NGF restricted to Schwan cells and mediated by axonal signals (Chan et al., 2004). In contrast, in a more recent study, NGF overexpression in human olfactory bulb NSCs boosts oligodendrocyte differentiation (Marei et al., 2013), while seeming to inhibit proliferation and OL differentiation in rodent NSCs in a process once again mediated by *p75^NTR^* (Guo et al., 2013).

The NGF dynamic regulation demonstrated in the present study throughout the entire differentiation process, might explain these contrasting results, at least in part. During *in vitro* OL maturation, *NGF* gene expression increases almost 60 times when OL lineage is established, but cells are still proliferating. This expression is also followed by an increase in NGF protein production and secretion in the culture medium, suggesting a key role of this factor in the differentiation process. Moreover, *TrkA* gene expression also increases during the first phase of differentiation, while, as expected, *p75^NTR^* is drastically decreased following oligodendrocyte lineage induction of the multipotent NSCs: indeed this gene is a recognized marker for undifferentiated neuroectodermal precursors (Moscatelli et al., 2009).

Our mixed system does not allow us to identify which cell type is expressing the gene of interest or secreting the NGF protein. However, we were able to establish that astrocytes are the main cells producing NGF protein, while PDGFαR-positive OPCs express both the TRKA and p75^NTR^ proteins in an undifferentiated state. The dynamic of *TrkA* gene expression in the culture and the TRKA protein expression in OPCs suggests that these cells may be a NGF target. It has been widely established since the first characterization of the NGF/TRKA interaction in 1990s (Miller et al., 1991; Holtzman et al., 1992; Li et al., 1995; Zhou et al., 1995) and in more recent studies (Calvo et al., 2015), that TRKA expression is directly regulated by NGF. Thus, to confirm that OPCs are not only possible NGF targets but they are able to efficiently respond to this growth factor action, we proved the TRKA upregulation as a result of exogenous NGF addition in the culture medium.

Taken together, all this data suggests that in a mixed cellular environment more closely resembling the *in vivo* condition, astrocytes produce NGF which acts on the OPC differentiation and maturation processes. To prove this hypothesis, we blocked the action of endogenous NGF using both a specific antibody anti-NGF and a specific TRKA antagonist, finding that sequestration of the secreted NGF in the culture medium or TRKA blocking impairs OPC differentiation/maturation, resulting in an increased percentage of undifferentiated precursors (OPCs, NG2-positive cells) and a dramatic decrease in the percentage of mature (CNPase-positive) and myelinating (MBP-positive) OLs. Therefore, in these experimental conditions NGF is required for proper OPC maturation from NSCs. In fact, exogenous NGF in control conditions does not affect spontaneous OPC cell death. This is in line with studies from other groups, demonstrating that the inhibition of NGF action in adult SVZ-derived NSCs impairs their proliferation and differentiation (Scardigli et al., 2014).

Our data contradict a recent paper reporting that NGF neutralization results in an increase in oligodendrogenesis of NSCs (Brandi et al., 2021). Substantial differences exist from this and our study. In the Brandi’s study NGF neutralization is assumed to be obtained deriving cells from mice expressing the recombinant version of the mAb αD11 NGF neutralizing antibody, but a characterization of the *in vitro* expression of the NGF neutralizing antibody is not presented. Thus, it is not clear if the NGF biological activity was actually inhibited at the *in vitro* level. Moreover, this study is performed using hippocampal-derived neurospheres, which is not the most suitable model for oligodendrocyte lineage studies. The hippocampal NSCs, in fact, are not prone to differentiate in the oligodendrocyte lineage, both *in vivo* and *in vitro*, compared to NSC derived from fetal forebrain used in our study, generating very few or none mature cells (Bonaguidi et al., 2011; Han et al., 2015). This is widely reported in the literature [reviewed by Kamen et al. (2022)]. In fact, in the study of Brandi et al. the oligodendrocyte differentiation in WT cultures is very low (less than 2% of O4-positive cells and 0% of MBP-positive cells), slightly increased in transgenic mice (less than 9% of O4-positive cells and less than 4% of MBP-positive cells) leading to a strong bias related to the model. As shown in the present study and as already published (Baldassarro, 2021), in our model, we obtained more than 70% of CNPase mature oligodendrocytes and 50% of the whole culture that was also positive for MBP. Even if the authors replicated the experiment on primary oligodendrocytes, they never reached more than 5% of mature MBP expressing cells. In fact, in both *ex vivo* models, the oligodendrocyte differentiation was not induced by T3 or any other physiological stimuli leading, again, to strongly biased results which are very far from the *in vivo* conditions. This was also reported in previous experiments texting NGF and derived molecules, carrying the same bias (Malerba et al., 2015).

If the role of NGF in OPC biology is disputed, its role during neuronal/axonal damage and demyelinating lesions and diseases in the neonatal and adult CNS becomes highly uncertain.

Extensive OPC and OL degeneration and the consequent impairment of myelination are major features of the brain damage observed in neonatal encephalopathy and consequent chronic neurological disabilities (Millar et al., 2017). We therefore focused on fetal OPCs, with the aim of establishing whether NGF protects these cells in a well-described model of OGD, one which mimics the HI and reoxygenation/reperfusion occurring in neonatal encephalopathies (Baldassarro et al., 2018). We have already shown how PARP inhibitors, putative neuroprotective molecules (Baldassarro et al., 2017a), are noxious to fetal but not adult OPCs (Baldassarro et al., 2018), thus undermining the neuroprotective role of these compounds in neonatal encephalopathies. Moreover, the glucose metabolism of OPCs derived from fetal and adult NSCs is substantially different (Baldassarro et al., 2019b). OGD in particular induces OPC death and an impairment of surviving cell differentiation (Baldassarro et al., 2019b). When applied at the beginning of the differentiation phase, it induces an increase in NGF gene expression, as already shown in Schwann cells (Zhu et al., 2010), indicating that NGF partially protects the OPC lineage from both cell death and differentiation impairment. This result suggests that NGF should be considered as a therapeutic agent for neonatal demyelinating diseases.

Moreover, given that the astrocyte seems to be responsible for NGF production, we showed that ACM, containing the astrocyte secretome which includes a significant amount of NGF (more than 1,000 times higher than OPC medium), is able to protect OPCs from OGD-induced cell death. In fact, astrocytes are known to secrete several growth factors (Cabezas et al., 2016), and respond to different CNS injuries, increasing the production of NGF (Goss et al., 1998; Cheng et al., 2019).

Since we demonstrated that blocking NGF action, and specifically TRKA signaling, impairs OPC survival and differentiation, we hypothesized that also the protection from OGD-induced cell death may occur through this receptor. In fact, TRKA activation by NGF protects cultured neurons from glucose levels fluctuation by activating the AKT pathway (Yan et al., 2020), a signal transduction path which is activated by NGF in different cell types (Geetha et al., 2013; Griffin et al., 2020). In these contexts, NGF seems to exert its action, by TRKA interaction, increasing the presence of the phosphorylated form of AKT in the nucleus (Xuan Nguyen et al., 2006).

Here we describe that soon after the OGD exposure in presence of NGF, both total AKT and phosphorylated active AKT levels increase in the OPCs nucleus, and the simultaneous presence of the TRKA inhibitor or the specific antibody blocking the NGF action in the culture media selectively blocks the pAKT increase.

It is widely described that AKT signaling is required for proper myelination and it is dysregulated in white matter abnormalities (Wang et al., 2022). It has also been described that AKT1/2/3 Knock-out mice lack mature OLs (Wang et al., 2021). Different molecules have been indicated as both AKT activators (e.g., neuregulin1, ErbB, and ECM proteins) and downstream regulators (e.g., mTOR1, mTOR2, TSC1/2, RHEB, and Sox10 through FoxO1) (Wood et al., 2013; Wang et al., 2022). In the present study we provide solid evidence that also NGF/TRKA pathway activates AKT during OPC differentiation/maturation. The detailed description of the downstream mediators deserves a wide and deep further investigation.

One current hypothesis regards the possible involvement of NGF in the demyelinating lesions and disease characteristics of several health conditions, such as multiple sclerosis and related animal models, where an increase in NGF production is described (Calzà et al., 1997; Micera et al., 1998). In addition to immune-suppressive/modulatory functions (Villoslada et al., 2000), a positive role in re-myelination and OPC and OL protection has been suggested in different demyelination models (Acosta et al., 2013). Our group also showed that in demyelinating conditions, there is an increased proliferation of *p75^NTR^*-positive cells in the SVZ, cells which have been recognized as OPCs by other research groups (Oderfeld-Nowak et al., 2009), which then migrate to the white matter of the corpus callosum (Calzà et al., 1998). Moreover, NGF protects OLs from TNFα-induced toxicity (Takano et al., 2000), an action which is not restricted to resident OPCs but extends to the precursors generated in the SVZ, the CNS stem cell niche (Petratos et al., 2004).

In conclusion, our results demonstrate a role of NGF in physiological OPC differentiation in an *in vitro* system modeling the interplay with astrocytes, the main NGF source in this culture. Moreover, its protective action with regard to OPC survival and OL maturation in the presence of strong noxious stimuli, known to be a key component in damage to both neuronal and myelin forming cells, corroborates its full neuroprotective function and suggests potential implications for the treatment of demyelinating diseases.

## Data availability statement

The original contributions presented in this study are included in the article/Supplementary material, further inquiries can be directed to the corresponding author.

## Ethics statement

The animal study was reviewed and approved by Aut. n. 635/2018-PR - 03 Sept 2018.

## Author contributions

LA, LC, and LG designed and coordinated the study. VAB, MC, LL, and MLR performed the experiments, acquired and analyzed the data. VAB and LC interpreted the data. VAB and LC interpreted the data and wrote the manuscript. All authors approved the final version of the manuscript.
