# Supplementary material for: Nerve growth factor promotes differentiation and protects the oligodendrocyte precursor cells from in vitro hypoxia/ischemia
Source: Front Neurosci. 2023 Feb 16;17:1111170. doi: 10.3389/fnins.2023.1111170 (PMC9978228; doi:10.3389/fnins.2023.1111170)
Supplement: Supplementary file 1 [file Data_Sheet_1.pdf]

SUPPLEMENTARY MATERIAL

Nerve growth factor promotes differentiation and protects the oligodendrocyte precursor cells from in vitro hypoxia/ischemia.

RESULT S1. Validation of the cell death quantification using the LDH assay

Methods S1.
LDH activity measurement was performed with PIERCE LDH Cytotoxicity Assay Kit, following manufacturer's instructions, using the supernatant of the described experimental group.

Results S1.
To validate the quantification of cell death by nuclear morphology analysis, we performed the LDH activity assay in the supernatant of the main experimental groups.
We quantified the cell death using the LDH activity, the enzyme released from the cell as the final result of the cell death process leading to the lysis of the cell membranes and the release of the cytoplasmatic content, containing the enzyme, in the culture medium. Thus, we used the OGD condition as a cell death positive control group (100%) to perform a semi-quantitative analysis as a comparison with all the other groups (Figure S1).
We analyzed the cell death in normoxia, in cultures treated with vehicle, as a cell death negative control, or with the two test conditions: GW-441756 (10 μM) or Ab-NGF (10 μg/ml). LDH assays confirmed the cell death induction due to the blocking of the NGF activity in physiological conditions (One-way ANOVA, $F_{(2,6)} = 51.93$, $p = 0.0002$) only when TRKA receptor is directly inhibited (Dunnett's post-test, GW-441756, $p = 0.0002$), as described by nuclear morphology analysis (Figure 3B).
Moreover, we tested the effect of exogenous NGF administration to the culture medium in OGD conditions mimicking the hypoxia/ischemia pathology. The LDH assay confirmed the protective effect of NGF described with nuclear morphology analysis (Figure 4), showing a decrease in the enzyme activity quantification (Student's t-test, $p = 0.0264$).

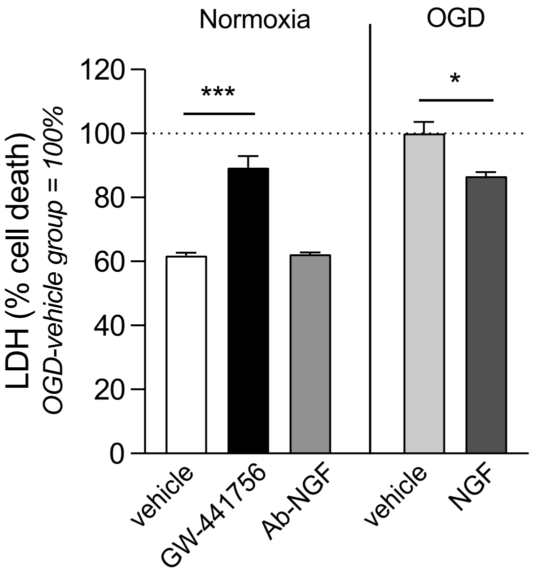

FIGURE S1. LDH assay.
Graph shows the quantification of cell death through the LDH assay in normoxia and OGD conditions. Data are expressed as percentage of the OGD-vehicle group as a positive cell death control (100 %; light gray bar and horizontal dotted line). Bars represent mean ± SEM. Statistical analysis: One-way ANOVA, followed by Dunnett's post-test (normoxia) or Student's t-test (OGD). Asterisks represent the differences between cultures the groups indicated with a horizontal line (* $p < 0.05$; *** $p < 0.001$).
Abbreviations: Ab-NGF, antibody anti-nerve growth factor; NGF, nerve growth factor; OGD, oxygen-glucose deprivation.

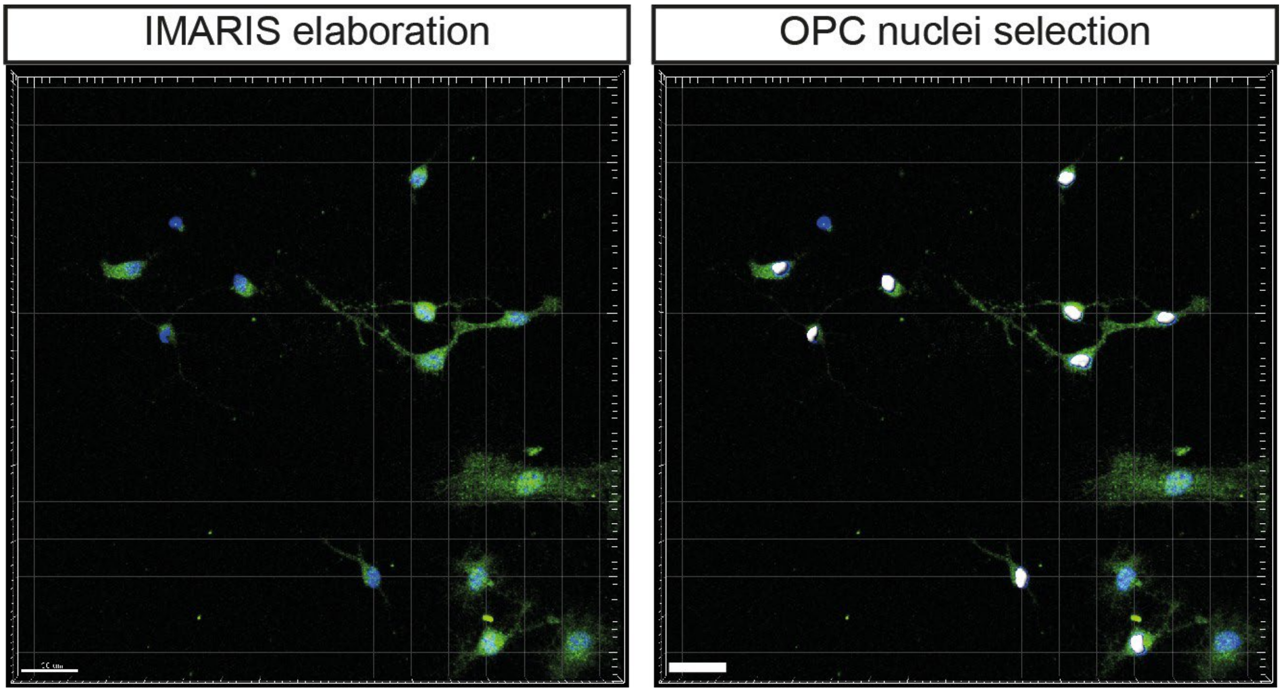

FIGURE S2. AKT/pAKT quantification using IMARIS software
Representative confocal images elaborated by IMARIS software. The reconstruction of nuclear isosurfaces was based on the nuclear staining, OPCs nuclei were selected based on nuclear size (smaller than astrocytes) and cell morphology (OPCs: small and round cell body with short ramifications; astrocytes: fibroblasts-like flat cell body). Fluorescence intensity of AKT or pAKT staining was measured inside the nuclear isosurfaces. Scale bar: 30 µm.

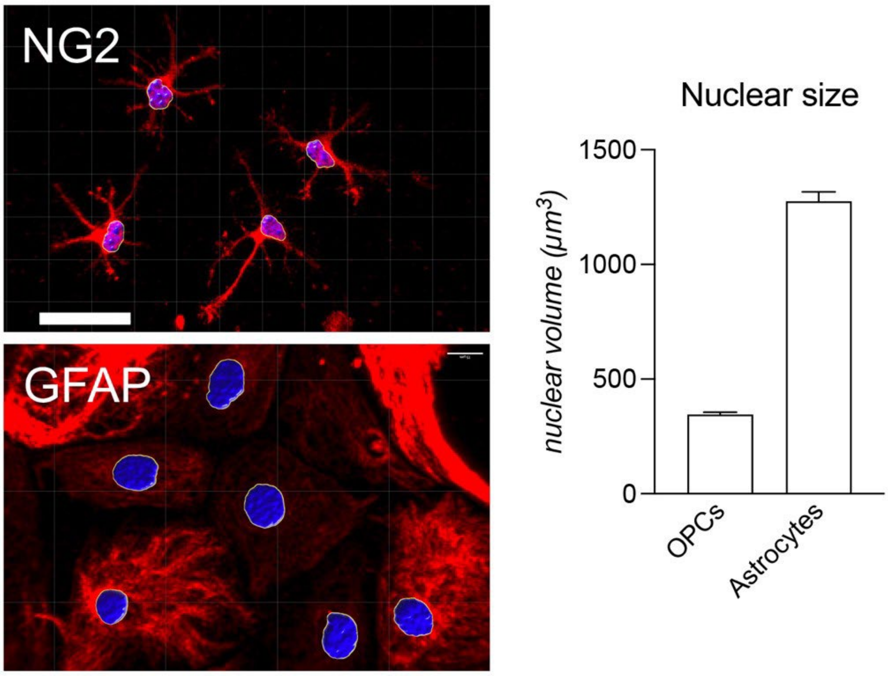

FIGURE S3. Quantification of the nuclear size in OPCs and astrocytes.
Representative images of IMARIS based software elaboration of the nuclei marked with Hoechst dye and reconstructed using isosurfaces in OPCs (NG2-positive cells) and astrocytes (GFAP-positive cells). Graph represents the average nuclear size of the two cell lineages (n = 100 cells per lineage). Scale bar: 30 μm.
